# Informal carers’ perception on perspective taking when reporting as proxies about someone else’s health

**DOI:** 10.1007/s11136-026-04375-w

**Published:** 2026-08-22

**Authors:** Henok Dagne, Kathleen Doherty, Alice Saul, Julie Campbell, Ingrid van der Mei, Bruce V. Taylor, Jessica Roydhouse

**Affiliations:** 1 Menzies Institute for Medical Research, University of Tasmania, Hobart, Australia; 2 Wicking Dementia Research and Education Centre, University of Tasmania, Hobart, Australia

**Keywords:** Proxy, Perspective, Dementia, Multiple sclerosis, Person-centred care

## Abstract

**Purpose:**

Informal carers (‘proxies’) are often asked to report on the health of individuals who cannot self-report, either from their own (‘proxy-proxy’) or the patient’s (‘proxy-person’) perspectives. It remains unclear which perspective proxies find most useful. Understanding this is important for designing measures that proxies can complete. We conducted an exploratory study on which perspective proxies find most useful and why, when reporting pain and physical function in dementia and symptoms in multiple sclerosis (MS).

**Methods:**

This study used data from two cross-sectional online surveys: one for proxies of people with MS and another for people with dementia. Proxies were asked to indicate which perspective they found most useful and why for communicating symptoms in MS, and pain or physical function in dementia, to healthcare professionals (HCPs). Quantitative responses were summarised using percentages; qualitative responses were analysed using content analysis.

**Results:**

55 proxies reported on MS symptoms, 29 on pain in dementia, and 33 on physical function in dementia. The most selected perspectives were: 35% (19/55) proxy-proxy for MS symptoms, 38% (11/29) proxy-person for pain and 39% (13/33) either of the perspectives for physical function. Proxy-person was valued for empathy and person-centredness, especially in reporting pain and MS symptoms. Proxy-proxy was appreciated for objectivity and reducing conflict, particularly in physical function. Using either perspective was considered most useful for balancing views and reducing misinterpretation in both conditions.

**Conclusion:**

Proxies’ most useful perspectives vary slightly by domain and condition. Incorporating an understanding of how proxies approach the challenge of reporting may improve measure design.

**Supplementary Information:**

The online version contains supplementary material available at https://doi.org/10.1007/s11136-026-04375-w.

## Abstract (plain language summary)

Questionnaires for informal carers (‘proxies’) typically ask them to report either from their point of view or from the viewpoint of the person with they care for. However, it is not clear which viewpoint proxies find most useful.

In this study, proxies were asked which viewpoint they considered as most useful when reporting on symptoms in multiple sclerosis, and pain and physical function in dementia. They were also asked to explain why they selected that viewpoint as most useful. The viewpoints the proxies found as most useful varied slightly by health conditions and domains. Most proxies selected reporting from their viewpoint as most useful for symptoms communication in multiple sclerosis, the persons’ viewpoints for pain in dementia, and either of the viewpoints for physical function in dementia. Proxies who selected person’s viewpoint as most useful perceived it to be more person-centred. Those who selected their own viewpoint explained that it helped them to provide more objective report.

Understanding more about how proxies approach reporting about someone else’s health, including the viewpoint that they use to do this, may offer some insights to improve how questionnaires that proxies complete are designed. For example, these insights could inform the development of clearer instructions, which may, in turn, support the quality of the information provided by proxies. However, given the exploratory and context-specific nature of the findings, these potential applications require further investigation.

**Supplementary Information:**

The online version contains supplementary material available at https://doi.org/10.1007/s11136-026-04375-w.

## Background

Proxy reporting involves obtaining information about patients’ health, including their health-related quality of life (HRQOL) or domains related to HRQOL. Proxy reporting is often sought if a patient has cognitive or physical impairments [1]. However, differences between proxy and patient reports are often raised as a concern when trying to interpret proxy-reported data [2, 3], including data about patient HRQOL.

Proxy reports are affected by several factors. This includes patient-related factors (e.g., disease severity, progression, educational status) [4], proxy-related factors (e.g., caregiving burden, age) [2, 4], and the proxy’s relationship with the patient [5–7]. The measure-related factors that can affect proxy reports include the observability of the domain to be reported [2], and the perspective the proxy are asked to adopt while responding [4, 8–10]. Although regulatory bodies such as the US Food and Drug Administration (FDA) [11] and the European Medicines Agency (EMA) [12] often view proxies as only taking one perspective when reporting, the literature describes more than one perspective: “proxy-person” (aligning with the regulatory definitions or view of proxies) and “proxy-proxy” [13]. The proxy-person perspective involves proxies reporting as they believe the patient would, adopting the patient’s viewpoint [14]. To illustrate this, an example question from the proxy-person perspective could be *“How much pain do you think the patient would say they are experiencing if they were able to communicate?”* In contrast, the proxy-proxy perspective reflects the proxy’s own assessment based on their observations and understanding. The previous question phrased using the proxy-proxy perspective could be *“How much pain do you think the patient is experiencing?”* Some proxy-reported outcome measures such as the Dementia Quality of Life (DEMQOL) [15] are designed from a proxy-person perspective, while others such as the Adult Social Care Outcomes Toolkit (ASCOT) [16] is designed to ask the proxy to consider both perspectives together [17]. The EQ-5D allows users to opt for one or both versions [18]. EuroQol, which is the developer of the EQ-5D, now provides only the proxy–proxy version, but the proxy‑person version is available by wording adjustment with permission [19].

Regardless of the perspective used, providing clear instructions to proxy respondents is recommended [1, 4]. The research objective should determine which perspective to use [20]. A qualitative study [21] proposed offering separate response options for each perspective to improve clarity and validity. The evidence on which perspective provides closer agreement with patient self-report is mixed. Some studies showed stronger agreement with self-report when proxy-person perspective is used [20, 22–25]. Another study [14] found better concordance with the proxy-proxy perspective for certain sub-scales. However, agreement of proxy-report with self-report is not the only metric used for evaluating proxy-report quality. Because self-reports are not available in the context where proxy reports are needed, such as advanced dementia, comparing proxy-reports with self-report is not always possible. This makes it problematic to rely on concordance as a primary quality indicator. Moreover, there is no evidence whether the proxies adhere to the perspective instructions provided [26].

Although evidence remains limited, some studies have examined how proxies interpret and follow perspective instructions for specific measures. For example, Rand and colleagues [16] explored proxies’ understanding of the proxy-proxy and proxy-person perspectives in the ASCOT-Proxy. Our study extends this literature by investigating interpretation and adherence across a broader range of domains relevant to HRQOL and does not focus on a single instrument. There is limited evidence on which perspective proxies may thought to be most useful to report about others’ health. This study, therefore, sought to identify which perspective proxies found most useful and why. This information may be helpful in guiding future research that can inform the design of future measures.

We aimed to gather this additional evidence in two different clinical contexts. The first was dementia, in which proxy report is widely used due to cognitive decline in the person living with dementia, especially in the later stages of the condition [27]. The second context was multiple sclerosis (MS), a context in which proxy reporting is less common, though not non-existent. In MS, proxy reporting may be used during mood disturbances, physical disability, or when physical impairments hinder self-reporting [28].

Additionally, given previous evidence that the observability of the domain on which the proxy reports may play a role in proxy reporting [2], we examined this across different HRQOL-related domains of varying observability. For example, less visible MS symptoms [29, 30] such as fatigue [31, 32], depression [33], pain and cognitive impairment [34, 35] and visible symptoms such as walking difficulty [29] significantly affect the HRQOL of the person with the condition [36]. Both pain [37] and physical function [38] are key determinants of HRQOL in people with dementia, but also differ in terms of observability. Our objective in this study was to undertake a preliminary study on which perspective proxies consider as most useful while communicating about the health of others to health care professionals (HCPs) and explore their reasons for this choice across these different domains and clinical contexts. The focus was not on the evaluation of a specific measurement tool. Therefore, to orient participants and aim for consistency in how they interpreted the questions, communication with HCPs was used as a guiding context throughout the surveys. Anchoring the questions to communication with HCPs gave a shared reference point that could help participants reflect on real interactions rather than answering hypothetically.

## Methods

### Design

This study draws on two separate cross-sectional online surveys conducted with informal carers in two different populations: (1) carers of people living with MS and (2) carers of people living with dementia. People with lived experience of caring (for people with dementia/ for people with MS) reviewed and commented on the study materials. Ethics approval was obtained from the University of Tasmania Human Research Ethics Committee (H0030743 approval date 24/6/2024 and amendment date 20/12/2024 for the MS study; H0030059 for the dementia study approved on 18/03/2024). This study is part of a larger PhD project. The Standards for Reporting Qualitative Research (SRQR) checklist was used for reporting (Table S1) [39].

### Recruitment and data collection

The surveys were advertised via the *Understanding MS* and *Understanding Dementia* massive open online courses (MOOC), hosted by the University of Tasmania. The MS survey was administered using LimeSurvey (September – November 2024). The dementia survey was administered using REDCap in May – June 2024. Eligible participants for the MS survey were current informal (unpaid) carers of people living with MS, aged 18 years and above. For the dementia survey, both current or previous informal (unpaid) carers of people living with dementia, aged 18 years and above and who live in Australia were included. While we included current and former carers of people living with dementia, we included only current carers of people living with MS. Dementia is a progressive, terminal condition, and former carers can provide valuable information about pain and physical function assessment and communication. MS often has a variable course of symptoms. Some are less visible to carers and individuals with MS usually retain cognitive function to self-report on their symptoms. The classification of carers as current and former are more developed in dementia research compared to in MS. As the MS Role Survey asked carers their current role, we included only current carers in the MS part of the survey.

The anonymous surveys were administered and advertised in English. Participants were advised that survey completion implied consent to participation and data use. Both platforms required participants to access the survey through secure links embedded in the course pages. No public posting of survey links occurred. In the dementia survey we used the response date and time as this was fully anonymous. For the MS survey we used the MOOC participant unique identification number to remove duplicate responses. To reduce participant burden, we have asked those who indicated interest to participate in research in the MS Role Survey, an existing research study that is separate to this project. The MS Role Survey asks MS MOOC participants how they identify (e.g., a person living with MS, or a carer, family member, or friend of someone with MS) themselves. The MS Role Survey functioned as a screening and routing mechanism within the MOOC‑based research design, linking initial general consent to the delivery of the appropriate, role‑specific surveys. First, it classified participants according to their role within the MS community, allowing the researchers to distinguish between people with MS and carers/family/friends. Second, it helped to link between the MOOC platform and the appropriate follow‑up survey, ensuring that participants were only invited to surveys that were relevant to their role.

We used online qualitative surveys with free text responses. Qualitative online surveys, although relatively novel and underutilised in qualitative research [40], have potential in qualitative research [41]. Previous studies have shown the feasibility of qualitative online surveys for capturing participants’ experiences and perspectives across diverse research context [42–44]. For example, an international online qualitative survey including participants from 57 countries was used to explore the perspective of people with MS about their attitude toward and experiences of lifestyle modification as part of self-management [45]. Thus, online qualitative surveys offer potential of obtaining information from wide range of participants, including participants who might otherwise avoid in-person research [46] and from large, diverse samples [47]. In turn, the broader reach may enhance the range of perspectives captured and improve the transferability of findings to contexts beyond the immediate study setting [41, 48]. In the MS study, the online survey helped us to obtain responses from several countries.

## Perspectives

### Perspective for MS symptom communication

Due to less being known about proxy reporting in MS, we first asked carers about how often they accompanied the person living with MS to healthcare consultations (response options were never, rarely, sometimes, often and always). Carers who at least rarely accompany the person living with MS to healthcare visits were asked how often they communicate with HCPs about MS symptoms (with similar response options: never, rarely, sometimes, often and always). To understand the most useful perspective, we asked carers who at least rarely communicate MS symptoms with HCPs: “When communicating MS symptoms to HCPs, please indicate which approach you think would be most helpful.” The response options were:

1. Imagining yourself in their shoes and answering from their perspective.
2. Answering from your perspective (your own viewpoint as an observer).
3. Either.
4. Neither.

Carers who selected option 1 or 2 or 3, were asked a follow-up open-ended question: “Please explain why you think the approach you have selected is most helpful for communicating.” We intentionally left the term “useful/helpful” open to carers’ interpretation to capture their understanding rather than impose a predefined definition. This allowed carers to consider usefulness from any perspective they deemed relevant, whether their own, the person they care for, or HCPs.

### Perspective for pain and physical function communication in dementia

Separate prompts were given for questions about pain and physical function. For pain, carers were first asked if they perceive the person they care for experienced pain. Only carers who indicated the person they cared for experiences/ed pain were asked a perspective question: “If you are asked a question about their pain by HCPs, please indicate which approach you think would be most helpful in communicating.”

For physical function, carers were asked if they communicated about the physical function of the person living with dementia and were then asked to select the most useful perspective. The response options were the same as in the MS survey. Participants who selected options 1, 2 or 3 were then asked a follow-up open-ended question to explain why they think the approach or approaches they have selected is or are most helpful. Due to an error in skip logic, carers were only asked the open-ended question for most useful perspective to communicate physical function if they selected options 1 or 2.

### Data collection tool

In addition to the information about perspectives, we collected sociodemographic information about informal carers. This included age, gender, country (for proxies of people with MS only, as the dementia survey was limited to people living in Australia), education, carer status (current/former for dementia carers), years of caring experience, relationship with the person and cohabitation status (for MS carers). Additionally, we collected information about dementia and MS types and years since diagnosis.

### Data analysis

A descriptive analysis was conducted to report the carer-perceived frequency and percentage of the most useful perspectives. The analysis of carers’ reasons for choosing a perspective as the most useful focused on describing and categorising the reasons provided using content analysis. Categories and subcategories were identified from the data and were not pre-determined in advance. HD, a PhD student, developed the analysis plan, created a codebook, defined coding rules, and analysed the data, with guidance and feedback from JR throughout. Discrepancies were resolved through discussion. Quotes were labelled with participant number (Pn) and condition (MS or dementia). KD, AS, JC, IV, and BT reviewed the data collection tool and manuscript. Regular meetings supported rigorous analysis and researcher reflexivity.

## Results

### Participant characteristics

**Fig. 1:**
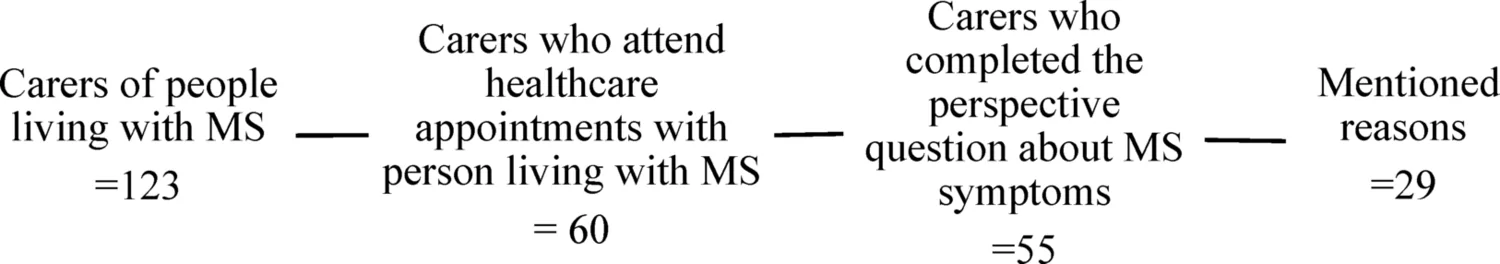
Flow chart of participants who answered the perspective questions in the MS survey

**Fig. 2:**
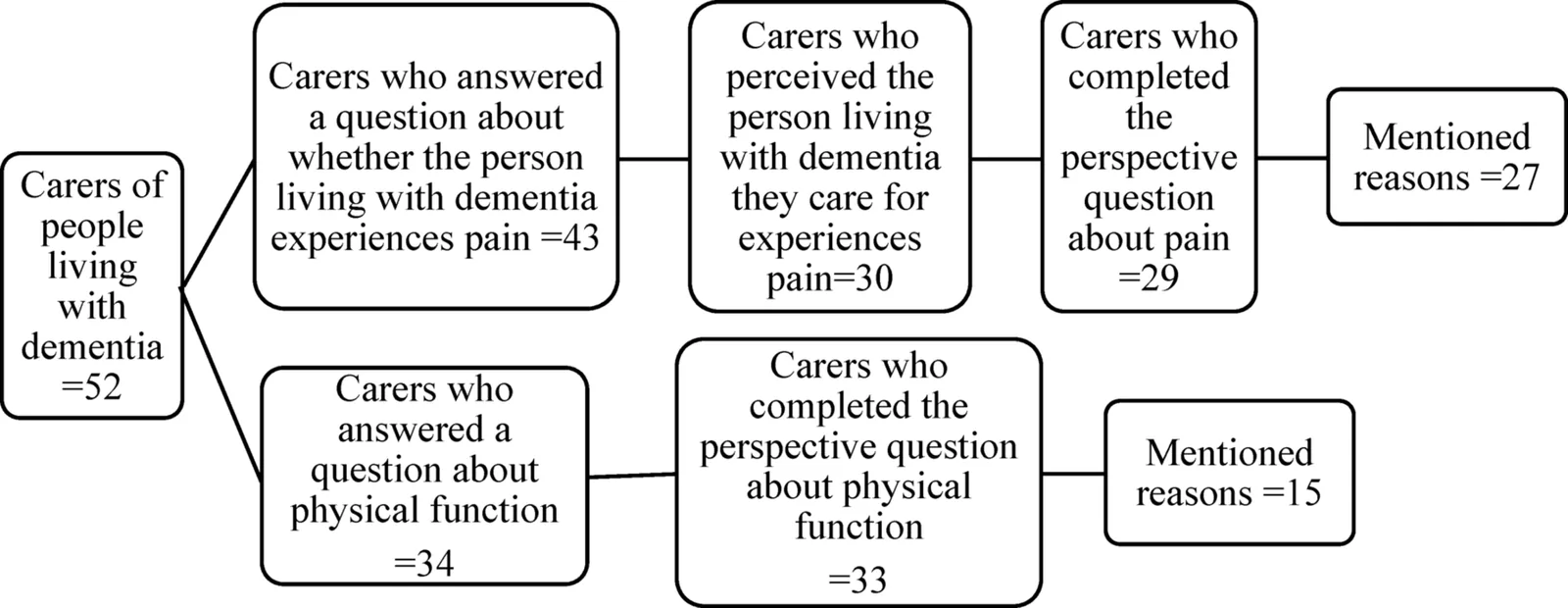
Flow chart of participants who answered the perspective questions in the dementia survey

A hundred and twenty-three carers of people living with MS participated in the MS survey. Out of these, 55 carers provided a response on which perspective they found most useful for communicating MS symptoms to HCPs, with 29 providing reasons for their choice (Fig. 1). In the dementia survey, 29 and 33 carers answered a question about the most useful perspective in pain and physical function communication, respectively (Fig. 2).

Most study participants in the MS survey were from Australia (27/55 or 53%), and the median age was 40 years. Most participants (35/55 or 66%) were women. (Table 1).

**Table 1:** Sociodemographic characteristics of carers of people with MS who completed the perspective question (*n* = 55)

Variables	Characteristic	For MS symptom report *n* (%)
Age in years	Median & IQR	40 (30–53)
Gender	Woman	35 (66%)
	Man	18 (34%)
Education	University-Postgraduate	10 (19%)
	Vocational/TAFE	16 (30%)
	University-undergraduate	15 (28%)
	Primary and secondary school	11 (21%)
	Prefer not to say	*
Currently living with the person with MS (*n* = 54)	Yes	22 (41%)
Carer experience in years	Median & IQR	2 (1–3)
Relationship with the person with MS	Parent	6 (11%)
	Child	7 (13%)
	Spouse	15 (27%)
	Another relative	10 (18%)
	Friend	5 (9%)
	Other	12 (22%)**
Type of MS onset	Progressive onset	14 (26%)
	Relapsing onset	24 (43%)
	Not sure	17 (31%)
Receive support for the tasks involved in caring for people with MS (*n* = 54)	Yes	13 (24%)
Countries (*n* = 51)	Australia	27 (53%)
	New Zealand	6 (12%)
	Asia^a^	8 (16%)
	Others^b^	10 (20%)

^a^United Arab Emirates, Bangladesh, India, South Korea, Kuwait, Uzbekistan, & Yemen; ^b^Other countries: United Kingdom, Ireland Canada, United States, Cuba, Egypt & Uganda; *Counts less than 5 have been suppressed **other relationship includes clients, support workers, employers

In the dementia survey, the median ages of the participants who answered the pain and physical function questions were 59 years and 61 years, respectively. Nearly all participants were women: *n* = 30 (91%) for the pain question and *n* = 25 (86%) for the physical function question (Table 2).

**Table 2:** Sociodemographic characteristics of carers of people with dementia who completed the pain or physical function questions

Variables	Characteristic	Pain question *n* (%)	Physical function question *n* (%)
Age in years	Median & IQR	59 (50–67)	62 (50–68)
Gender	Woman	25 (86%)	30 (91%)
	Man	3 (10%)	3 (9%)
	Non-binary	*	–
Education	University-Postgraduate	8 (28%)	11 (33%)
	Vocational/TAFE	6 (21%)	9 (27%)
	University-undergraduate	8 (28%)	7 (21%)
	Secondary school	7 (24%)	6 (18%)
	Prefer not to say	–	–
Carer category	Current carer	15 (52%)	19 (58%)
Carer experience in years	Median & IQR	4 (3–8)	4 (2–6)
Relationship	Parent	5 (17%)	10 (30%)
	Child	8 (28%)	7 (21%)
	Spouse	4 (14%)	6 (18%)
	Other relative	9 (31%)	8 (24%)
	Other	*	*
Type of dementia	Alzheimer’s disease	11 (38%)	14 (44%)
	Vascular dementia	6 (21%)	6 (19%)
	Mixed dementia	9 (31%)	9 (28%)
	Other	*	*
Receive support for the tasks involved in caring for person with dementia	Yes	19 (65%)	20 (61%)

*Counts less than 5 have been suppressed

### Carer perception of the most useful perspective

For communicating pain in dementia, 38% (11/29) of carers perceived the proxy-person perspective as the most useful, followed by either (*n* = 10, 35%). The results were slightly different for physical function in dementia, with 39% (13/33) of carers reporting that either of the perspectives was most useful. The same number of carers (*n* = 9, 27%) found the proxy-person and proxy-proxy perspectives to be the most useful for this domain. For MS symptom communication, the proxy-proxy perspective was considered the most useful perspective by 35% (19/55) of carers. Similar percentages of proxies for people with MS found either perspective (*n* = 15, 27%) and the proxy-person perspective (*n* = 13, 24%) to be the most useful. Across all conditions, few participants selected “neither” perspective as the most useful (Table 3).

**Table 3:** Perceived most useful perspectives by informal carers when communicating to HCP

	Frequency (%)				
	Proxy-person	Proxy-proxy		Either	Neither
MS symptoms (*n* = 55)	13 (24%)	19 (35%)	15 (27%)		8 (15%)
Dementia-pain (*n* = 29)	11 (38%)	7 (24%)	10 (35%)		1 (3%)
Dementia-physical function (*n* = 33)	9 (27%)	9 (27%)	13 (39%)		2 (6%)

### Proxies’ reasons for selecting a perspective as most useful

In our content analysis, we categorised the proxies’ perceived reasons into person-centred, proxy-centred, and communication-centred (Table S2). Person-centred reasons (e.g., not excluding the patient, person-first perspective) were cited when carers selected the proxy-person perspective as the most useful perspective. Proxy-centred reasons (e.g., providing additional or complementary insights, legitimacy, ability to add observations) were mentioned as reasons for choosing the proxy-proxy perspective. Communication and relationship factors (e.g., helping to communicate with HCPs, avoiding conflict, conveying objective insight) were cited for both proxy-person and proxy-proxy perspectives. *“Familiarity with the person”* was one of the reasons cited for either proxy-person or proxy-proxy perspectives across all domains (Table 4).

**Table 4:** Reasons provided by carers for selecting a perspective as most useful for communicating to HCPs

	Perspectives	Reasons*	Category of reason
MS symptom	Proxy-person	Familiarity with the person	Person-centred and proxy-centred
		Person-first perspective	Person-centred
	Proxy-proxy	Alternative objective insight	Communication-centred
		Carers able to add more observation on what the people with MS might miss	Person-centred and proxy-centred
		Carers don’t think they can report from the proxy-person perspective	Person-centred and proxy-centred
		Additional insights and usefulness of added perspective	Person-centred and proxy-centred
	Either	Complementary view	Person-centred
		Familiarity with the person	Person-centred and proxy-centred
Dementia-pain	Proxy-person	Familiarity with the person	Person-centred and proxy-centred
		Helps the person feel included	Person-centred
		Encourages the carers to be more engaged	Proxy-centred
		Person-first perspective	Person-centred
	Proxy-proxy	Helps carers to convey alternative objective insight	Proxy- centred and communication- centred
		Familiarity with the person	Person-centred and proxy-centred
		Helps to effectively communicate with HCPs	Proxy- centred and communication-centred
	Either	Balanced perspective	Proxy-centred
		Familiarity with the person	Person-centred and proxy-centred
		Communication and observation	Proxy-centred and communication- centred
Dementia-physical function	Proxy-person	Person-first perspective	Person-centred
	Proxy-proxy	Avoid conflict	Communication-centred
		Familiarity with the person	Person-centred and proxy-centred
		Alternative objective insight	Communication-centred
		Carers don’t think they can report from the proxy-person perspective	Proxy-centred and communication-centred

*The reasons and categories were developed from proxies’ responses.

### Reasons for perspective choice for MS symptom communication

#### a. Proxy-person perspective

Carers who selected this perspective as most useful cited familiarity with the person as a reason. As one carer mentioned, “*I am with my daughter a lot*,* so she communicates with me very well on how her symptoms are and how they affect her*” (P116, MS). Another viewed this perspective to be person-first, “*Not sure if it is more helpful*,* but seems more legit to centre the person with MS”* (P 231, MS). Interestingly, one carer misinterpreted the perspective as adopting the viewpoint of HCPs. “*When communicating symptoms of Multiple Sclerosis (MS) to healthcare professionals*,* it can be most helpful to imagine yourself in their [HCPs] shoes…*’’ (P243, MS).

#### b. Proxy-proxy perspective

Some carers who selected this perspective as most useful viewed it as more objective. For example, a carer stated, “*I can provide an objective account of the patient’s symptoms*,* uninfluenced by their personal biases or emotional state*.” (P140, MS).

Other carers selected this perspective due to uncertainty about speaking from the perspective of the person living with MS, in contrast to being able to speak from their own perspective.

“I can’t know what my partner is feeling but I can accurately describe what I see.” (P247, MS).

“I don’t know what it feels like so wouldn’t be accurate for me to speak from their point of view. As an observer I have insight they don’t have.” (P237, MS).

Other carers felt that since the person with MS could self-report, their role was to add an external viewpoint. A carer, for example mentioned *“I think the [person with MS] is best placed to describe their perspective of symptoms; an outsider perspective might be useful to give the healthcare professional a different point of view.”* (P169, MS). Another carer added, *“Because they’re already receiving her perspective … an outside perspective could be helpful.” (*P79, MS).

#### c. Either of the perspectives

Some carers found that either perspective supported a complementary view of the person’s symptoms. As one carer put it: *“Considering the individual’s experience …and also speaking from an outside perspective is important …”* (P184, MS). Other carers felt that their familiarity with the person enabled them to report from either perspective. A carer explained, *“I am with my daughter a lot and she communicate well with me about how she feels so I can communicate both ways”* (P116, MS). A quote from another respondent further clarifies this *“I do take daily notes… I can often recognise warning signs …the person often says*,* ‘you know me so well’”* (P204, MS).

### Reasons for perspective choice for pain communication in dementia

#### a. Proxy-person perspective

Some carers who selected this viewpoint saw the proxy-person perspective as a person-first perspective, emphasising that the individual’s experience should be prioritised. A carer explained, *“Her experience of pain rather than mine was most relevant”* (P32, Dementia).

Some carers believed their familiarity with the person as an important reason for choosing this perspective, believing it allowed them to better represent the patient’s experience as exemplified in a carer’s quote *“I knew my mother well and was able to put myself in her shoes…”* (P32, Dementia).

Additionally, carers noted that using the proxy-person perspective helped the person with dementia feel included. For example, a carer mentioned, “*They [people with dementia] need to feel as part of the present and not to ignore them”* (P51, Dementia). Other carers chose this perspective for reasons that supported them when reporting as a proxy or being a carer. For example, one carer stated “*It [proxy-person perspective] assists you to exert more effort in helping to master basic things*” (P51, Dementia). Another carer stated, *“Putting ourselves in their shoes makes it real in the sense of what exactly the person living with dementia is going through in pain”* (P33, Dementia).

#### b. Proxy-proxy perspective

Some carers chose this perspective because they perceived it allowed them to provide alternative and objective insights that the person with dementia might not be able to express. As one carer explained, “*Because you see what they can’t or won’t …*” (P40, Dementia), while another observed, “*We do not know what the person is thinking*” (P46, Dementia). This perspective was seen as more factual and less reliant on assumptions, with one carer stating, “*facts are better than guessing*” (P53, Dementia).

Carers also drew on their familiarity and close relationships with the person to inform their reports, as described by one participant: “*Close long-term relationship*,* could only imagine what Dad thought and felt.*.” (P30, Dementia).

Additionally, the proxy-proxy perspective was perceived as effective for communicating with HCPs. One carer noted, *“Because I believe I can explain her pain in a way that her Doctor believes….”* (P2, Dementia). Another highlighted the ability to use the person’s own words to facilitate understanding: *“I can potentially quote Dad using his words to help them find the source of the pain.”* (P3, Dementia). Carers also felt that this perspective enabled them to advocate effectively on behalf of the person, as reflected in the statement, *“…can communicate it [pain of Person living with dementia] effectively on their behalf [to HCPs]”* (P40, Dementia).

#### c. Either of the perspectives

Some carers who selected “either” further clarified that relying on a single perspective could lead to misinterpretation or incomplete understanding. They perceived considering either their own observations and the imagined experience of the person with dementia leads to a more comprehensive and empathetic understanding. A carer noted,

If I answer from my perspective as observer, I may interpret her pain incorrectly so if I also imagine it in her shoes, I could get a more balanced view (P35, Dementia).

Another described these perspectives as “*more empathetic / humane approaches*,* plus cover both areas of experience …*” (P36, Dementia). Another carer added “*Dementia is a very quick-thinking role for the caregiver … techniques are dynamic*” (P17, Dementia).

Carers who selected either of the perspectives as most useful also recognised the importance of familiarity with the person to report both ways. For example, a carer justified as: “*Probably only because I know my Dad better than most people*” (P13, Dementia).

Finally, carers who selected either of the perspectives as most helpful for reporting the pain experienced by the person with dementia perceived that reporting either of the ways is important for clearly communicating with HCPs as it enhances communication and observation. One carer explained:

Imagining the pain from Mum’s perspective helps me to properly acknowledge her pain and give it a voice. Then relaying to the health care provider (HCP) my observations help clarify the picture. This process helps Mum feel heard and believed, while also relaying the true situation to the healthcare professionals without undermining Mum (P11, Dementia).

### Reasons for perspective choice for physical function communication in dementia

#### a. Proxy-person perspective

Some carers selected this perspective as they felt it prioritised the need of the person with dementia and was person-first. A carer, for instance, noted *“…Putting myself in my partner’s shoes helps to see it from his perspective…”* (P24, Dementia). Another carer noted, *“They [Proxy-person perspectives] are best because they are patient-centred.”* (P51, Dementia).

#### b. Proxy-proxy perspective

Some carers chose this perspective for reasons related to their relationship with the person living with dementia. For example, a carer shared, “*Dad could be belligerent… He would get angry if I answered for him or on his behalf*.” (P30, Dementia).

Similar to the findings for selecting for the proxy-proxy perspective as the most useful for reporting about pain in dementia, other carers chose this perspective as they felt that they could not report from the proxy-person perspective. A carer for example wrote “*I lack the understanding of what being in Dad’s shoes would be like*.” (P3, Dementia). Another carer added *“I’ve tried to imagine what my husband’s experience … I can’t do it…”* (P14, Dementia).

## Discussion

Proxies’ perceived most useful perspective when reporting on different HRQOL-related domains varied modestly across and within conditions. One explanation for this heterogeneity could be that carers may have interpreted the question differently. Proxies provided different reasons for perceiving a perspective or perspectives as most helpful, including person-centred, proxy-centred and communication-centred reasons. Regardless of the perspective or the domain, familiarity with the person emerged as a shared rationale.

This variation shows that there is no one- size-fits-all approach. The number of carers who selected a perspective as most useful varied based on both the condition of the person for whom they were caring and the type of information the carer reported. In MS, especially in early and mid-stages, proxies may not need to imagine the person’s internal experience as people with MS could self-report. Cognitive impairment in MS tends to be slower, domain-specific, and less global than in dementia, although differentiating the impairment at severe levels is difficult [49]. People with MS may be able to reliably self-report even with cognitive symptoms [50]. In this context, proxies may lean toward the proxy-proxy perspective, relying on their own observations to supplement the person’s input and provide additional information. The variation on most useful perspective based on type of information reported may have implication for multidimensional HRQOL measure design.

The reasons for perceived usefulness of different perspectives in our study align with the themes identified by Caiels et al. [21]. Caiels et al. found that proxies’ preferences are influenced by what they consider beneficial for the person, what aligns with their own understanding, and what they believe facilitates better communication. Similarly, Lobchuk et al. [10] classified carers’ responses to perspective-taking prompts into categories such as patient-oriented and caregiver-oriented thoughts, which align with our findings of person-centred and proxy-centred reasons, respectively. The third category, communication-centred in our study reflects carers’ consideration of how best to convey information to HCPs. Lobchuk et al. similarly highlighted communication as relevant to carers’ interpretation of patients’ experiences, although their study focused on perspective-taking and communication difficulties rather than on how carers communicated information to HCPs [10]. These underlying rationales could be considered when designing or interpreting HRQOL or other patient-reported outcome measures. An important point, however, to consider is that the choice of perspective would be determined by the study purpose. For example, in clinical trials where proxy responses may be used to substitute for missing patient-reported outcomes, the proxy-person perspective may be used if it provides closer agreement with self-report as the aim is to reflect the patient’s own viewpoint [13]. This approach supports substitutability and maintains consistency with self-report measures. In contrast, if the goal is to provide an additional, complementary viewpoint, the proxy-proxy perspective may be more suitable. Clear guidance on which perspective to adopt may therefore be tailored to the purpose of measurement. Along with this, as explained in the conceptual model by Pickard and Knight, researchers should be aware that proxies could understand and respond to questions differently due to the perspective used, and this has implications for measurement validity and reliability. Clarifying why the researcher wanted a proxy respondent to report from a certain proxy perspective could help the respondents to follow the instructions [13].

A critical issue is whether proxies understand and follow the perspective provided in the instructions. Previous studies [21, 51] found that proxies may unintentionally switch perspectives unless explicitly reminded, pointing to the importance of clear instructions. As proxies may try to respond from the perspective or mix of perspectives that they find useful when answering questions about someone else’s health, clearly communicating the intended perspectives to proxies may help reduce misinterpretations and support adherence to instructions [16].

Our findings show that “familiarity with the person” was a rationale for both proxy perspective choices aligns with and extends prior research. For example, carers have emphasised that proxies should know the person well [21]. Similarly, the Health and Retirement Study [52] identified familiarity as an important characteristic of proxy respondents. Prior research [2] has suggested that familiarity can be operationalised through factors such as the intensity and frequency of contact, time spent together, and the quality of communication and relationship closeness. Relationship closeness, in particular, has been shown to reduce judgment error in proxy reports [53], and proxies with better knowledge of the person tend to provide responses that more closely align with self-reports [54]. Our findings show that the use of familiarity as a justification for perspective choice may vary by context. For the proxy-person perspective, familiarity was mentioned by proxies in the context of knowing the person to represent their lived experience. For the proxy-proxy perspective, familiarity may be used to justify providing an objective or complementary viewpoint. However, there is an underlying commonality that familiarity is an important justification provided by carers, regardless of the perspective selected as most useful for communicating with HCPs.

### Implications

These findings may have practical implications for improving proxy-reported measurement, including measurement of HRQOL or related domains. However, further investigation will be required before reaching conclusions about the generalisability and applicability of these findings to those contexts. The evidence from this exploratory study may help inform such future work. Our findings do not provide support for the suggestion that one specific perspective should be chosen for all conditions or domains. One possible approach could be designing measures that include both perspectives, an approach that is taken for ASCOT [17], which has been shown to be feasible and improve acceptability [16]. However, there are potential implementation challenges, such as determining which perspective to prioritise for decision-making and reporting if responses differ across perspectives. In any case, it is important to document which perspective was used, for example by asking proxies how or if they adhered to the specified perspective(s). The recommendation for documenting perspectives has previously been suggested by the International Society of Quality of Life Proxy Task Force [1]. Our findings about the importance of familiarity as a reason for perspective choice suggests that this should be captured as well when proxy reporting is used.

#### Limitations and strength

This study’s limitations include the use of self-reported online data from self-selected participants, which may introduce biases such as self-selection, social desirability, and voluntary response bias. In addition, the study sample size was relatively small. Web-based data collection excluded individuals without internet access. Future studies with more diverse samples may generate additional or different insights. We used free text online qualitative surveys instead of qualitative focus group or interview, which limited the opportunity to clarify concepts to respondents or to probe responses in depth. At the same time, the online survey approach supports collecting information from diverse populations [42–44], makes surveys accessible to hard-to-reach participants [46], and facilitates anonymous [46] and rapid data collection. Future research may be able to add to this work by examining if respondents have fixed views of ‘useful’ perspectives or if they change their responses regarding ‘useful’ perspectives when probed.

The current study focusses on MS symptoms in general, and symptoms may vary in terms of observability, as well as pain and physical function in dementia. Future studies could consider other aspects of HRQOL such as independence, control, social participation, and emotional function. Although this study did not assess the perceived most useful proxy perspective in the context of completing a specific HRQOL instrument, its findings could inform future work focusing on specific HRQOL or PRO instruments. Additionally, only current carers were included in the MS survey, and the classification of current and former carer was left open for respondents’ interpretation in the dementia survey. We did not include this in the analysis. We acknowledge that asking carers about past situations could add to the complexity of proxy reporting. Future studies could consider the effects of asking carers about past events and the effect of recall period on proxy reporting. The current study was a preliminary online survey with a small sample size. Further studies with other qualitative techniques may be helpful. Lastly, entry codes and passwords were not used to ensure data quality in both surveys.

Nonetheless, this preliminary study had several strengths. In particular, study materials were reviewed by people with lived experience of being a carer. The study also provides additional evidence in an area in which evidence is scarce, helping to build the groundwork that can support improved measure design and administration of proxy-reported measures when HRQOL or related domains are assessed.

## Conclusion

Proxies’ choices for perspective-taking varied by health condition and the type of information reported. However, regardless of the chosen perspective, familiarity with the person with the condition was an important reason for choosing a perspective. Proxies selected perspectives as useful based on their understanding about the perspective’s advantage for the person, themselves or the communication process. Understanding how proxies approach reporting about someone else can help improve proxy reporting.

## Supplementary Information

Below is the link to the electronic supplementary material.

Supplementary Material 1

## Data Availability

Data is provided within the manuscript or supplementary information files.

## References

[1] Lapin, B., Cohen, M. L., Corsini, N., Lanzi, A., Smith, S. C., Bennett, A. V., Mayo, N., Mercieca-Bebber, R., Mitchell, S. A., Rutherford, C., & Roydhouse, J. (2023). Development of consensus-based considerations for use of adult proxy reporting: An ISOQOL task force initiative. *Journal of Patient-Reported Outcomes*, *7*, 54. 10.1186/s41687-023-00588-6 37266745 PMC10238331

[2] Lopez, A., Tinella, L., Caffò, A., & Bosco, A. (2023). Measuring the reliability of proxy respondents in behavioural assessments: an open question. *Aging Clinical and Experimental Research*, *35*(10), 2173–2190. 10.1007/s40520-023-02501-z 37540380 PMC10520105

[3] Hack, T. F., McClement, S. E., Chochinov, H. M., Dufault, B., Johnston, W., Enns, M. W., Thompson, G. N., Harlos, M., Damant, R. W., & Ramsey, C. D. (2018). Assessing symptoms, concerns, and quality of life in noncancer patients at end of life: How concordant are patients and family proxy members? *Journal of Pain and Symptom Management*, *56*(5), 760–766. 10.1016/j.jpainsymman.2018.07.019 30076964

[4] Rand, S., & Caiels, J. (2015). *Using proxies to assess quality of life: a review of the issues and challenges* (Discussion Paper No. 2899). Quality and Outcomes of person-centred care policy Research Unit (QORU), University of Kent. https://kar.kent.ac.uk/55009/

[5] Magaziner, J., Bassett, S. S., Hebel, J. R., & Gruber-Baldini, A. (1996). Use of proxies to measure health and functional status in epidemiologic studies of community-dwelling women aged 65 years and older. *American Journal of Epidemiology*, *143*(3), 283–292. 10.1093/oxfordjournals.aje.a008740 8561163

[6] Pierre, U., Wood-Dauphinee, S., Korner-Bitensky, N., Gayton, D., & Hanley, J. (1998). Proxy use of the Canadian SF-36 in rating health status of the disabled elderly. *Journal of Clinical Epidemiology*, *51*(11), 983–990. 10.1016/S0895-4356(9800090-0) 9817116

[7] Wolinsky, F. D., Ayres, L., Jones, M. P., Lou, Y., Wehby, G. L., & Ullrich, F. A. (2016). A pilot study among older adults of the concordance between their self-reports to a health survey and spousal proxy reports on their behalf. *BMC Health Services Research*, *16*, 485. 10.1186/s12913-016-1734-6 27612571 PMC5017056

[8] Lobchuk, M. M., McClement, S. E., Daeninck, P. J., Shay, C., & Elands, H. (2007). Asking the right question of informal caregivers about patient symptom experiences: multiple proxy perspectives and reducing interrater gap. *Journal of Pain and Symptom Management*, *33*(2), 130–145. 10.1016/j.jpainsymman.2006.07.015 17280919

[9] Egan, P. (2020). *Perspective Taking by Dementia Family Caregivers: A multi-method study of how proxy reporters assess quality of life in dementia [Doctoral dissertation, University of Wisconsin–Madison]*. University of Wisconsin–Madison Libraries. https://search.library.wisc.edu/digital/ARXCO5CWNQF2KQ8Q

[10] Lobchuk, M. M., McClement, S. E., Daeninck, P. J., & Elands, H. (2007). Caregiver thoughts and feelings in response to different perspective-taking prompts. *Journal of Pain and Symptom Management*, *33*(4), 420–433. 10.1016/j.jpainsymman.2006.09.021 17397703

[11] U. S. Food & Drug Administration. (2009). *Guidance for industry: Patient-reported outcome measures- Use in medical product development to support labeling claims*. U.S. Department of Health and Human Services.

[12] European Medicines Agency (2016). *Appendix 2 to the guideline on the evaluation of anticancer medicinal products in man: The use of patient-reported outcome measures in oncology studies.*

[13] Pickard, A. S., & Knight, S. J. (2005). Proxy evaluation of health-related quality of life: A conceptual framework for understanding multiple proxy perspectives. *Medical Care*, *43*(5), 493–499. 10.1097/01.mlr.0000160419.27642.a8 15838415 PMC1188232

[14] Gundy, C. M., & Aaronson, N. K. (2008). The influence of proxy perspective on patient-proxy agreement in the evaluation of health-related quality of life: an empirical study. *Medical Care*, *46*(2), 209–216. 10.1097/MLR.0b013e318158af13 18219250

[15] Smith, S., Lamping, D., Banerjee, S., Harwood, R., Foley, B., Smith, P., Cook, J., Murray, J., Prince, M., & Levin, E. (2007). Development of a new measure of health-related quality of life for people with dementia: DEMQOL. *Psychological Medicine*, *37*(5), 737–746. 10.1017/S0033291706009469 17176501

[16] Rand, S., Caiels, J., Collins, G., & Forder, J. (2017). Developing a proxy version of the adult social care outcome toolkit (ASCOT). *Health and Quality of Life Outcomes*, *15*, 108. 10.1186/s12955-017-0682-2 28526055 PMC5438504

[17] Rand, S., Caiels, J., Silarova, B., Towers, A. M., & Welch, E. (2025). *Adult Social Care Outcomes Toolkit Proxy (ASCOT-Proxy) Guidance*. University of Kent.

[18] EuroQol Research Foundation (2025). Proxy. https://euroqol.org/proxy-2/

[19] EuroQol Research Foundation (2025). Notice on changes to Proxy2 availability.

[20] Engel, L., Sokolova, V., Bogatyreva, E., & Leuenberger, A. (2024). Understanding the influence of different proxy perspectives in explaining the difference between self-rated and proxy-rated quality of life in people living with dementia: A systematic literature review and meta-analysis. *Quality of Life Research*, *33*(8), 2055–2066. 10.1007/s11136-024-03660-w 38656407 PMC11286712

[21] Caiels, J., Rand, S., Crowther, T., Collins, G., & Forder, J. (2019). Exploring the views of being a proxy from the perspective of unpaid carers and paid carers: Developing a proxy version of the adult social care outcomes toolkit (ASCOT). *BMC Health Services Research*, *19*, 201. 10.1186/s12913-019-4025-1 30922307 PMC6440097

[22] Leontjevas, R., Teerenstra, S., Smalbrugge, M., Koopmans, R. T., & Gerritsen, D. L. (2016). Quality of life assessments in nursing homes revealed a tendency of proxies to moderate patients’ self-reports. *Journal of Clinical Epidemiology*, *80*, 123–133. 10.1016/j.jclinepi.2016.07.009 27475879

[23] McPhail, S., Beller, E., & Haines, T. (2008). Two perspectives of proxy reporting of health-related quality of life using the Euroqol-5D, an investigation of agreement. *Medical Care*, *46*(11), 1140–1148. 10.1097/MLR.0b013e31817d69a6 18953224

[24] Hutchinson, C., Whitehurst, D. G., Crocker, M., Lay, K., Engel, L., & Ratcliffe, J. (2023). Measuring quality of life in residential aged care using the EQ-5D-5L: A cross-sectional study on the impact of cognition level and proxy perspective on interrater agreement. *Health & Social Care in the Community*, *2023*, Article5839776. 10.1155/2023/5839776

[25] Kuharic, M., Mulhern, B., Sharp, L. K., Turpin, R. S., & Pickard, A. S. (2024). Understanding caregiver burden from multiple perspectives: Dyadic agreement between caregiver and care recipient. *Quality of Life Research*, *33*(6), 1719–1734. 10.1007/s11136-024-03643-x 38632146

[26] Dagne, H., Doherty, K., Campbell, J., Saul, A., & Roydhouse, J. (2025). Proxy reporting in health: A scoping review of instructions, perspectives, and reporting experiences. *Quality of Life Research*, *34*(7), 1835–1847. 10.1007/s11136-025-03929-8 40011354 PMC12182471

[27] Bulamu, N. B., Kaambwa, B., & Ratcliffe, J. (2015). A systematic review of instruments for measuring outcomes in economic evaluation within aged care. *Health and Quality of Life Outcomes*, *13*, 179. 10.1186/s12955-015-0372-8 26553129 PMC4640110

[28] van der Linden, F. A., Kragt, J. J., van Bon, M., Klein, M., Thompson, A. J., van der Ploeg, H. M., Polman, C. H., & Uitdehaag, B. M. (2008). Longitudinal proxy measurements in multiple sclerosis: Patient-proxy agreement on the impact of MS on daily life over a period of two years. *BMC Neurology*, *8*, 2. 10.1186/1471-2377-8-2 18307788 PMC2270863

[29] Zhang, Y., Taylor, B. V., Simpson Jr, S., Blizzard, L., Campbell, J. A., Palmer, A. J., & van der Mei, I. (2021). Feelings of depression, pain and walking difficulties have the largest impact on the quality of life of people with multiple sclerosis, irrespective of clinical phenotype. *Multiple Sclerosis Journal*, *27*(8), 1262–1275. 10.1177/1352458520958369 32924841

[30] Sander, L., Kugler, J., & Elsner, B. (2020). The influence of multiple sclerosis-related symptoms on health-related quality of life. *Fortschritte der Neurologie-Psychiatrie*, *88*(11), 704–712. 10.1055/a-1113-7702 32356284

[31] Alharbi, A. (2025). Assessment of quality of life and fatigue in patients with multiple sclerosis-a cross-sectional study. *Journal of Pioneering Medical Sciences*, *14*, 440–448.

[32] Al-Gamal, E., Hyarat, S. Y., Jaried, A., Rama, L., Ahmad, E., M., & Long, T. (2025). Fatigue and health-related quality of life in patients with multiple sclerosis. *Journal of Research in Nursing*, *30*(2), 141–154. 40191838 10.1177/17449871241290435 PMC11969484

[33] Aparicio-Castro, E, Candeliere-Merlicco, A., Santa, C., & Villaverde-González, R. (2025). Association of depression in multiple sclerosis with fatigue, sleep disturbances, disability, and health-related quality of life: Outcomes of a cross-sectional study. *Neurology Perspectives*, *5*(1), 100181. 10.1016/j.neurop.2024.100181

[34] Bergmann, C., Becker, S., Watts, A., Sullivan, C., Wilken, J., Golan, D., Zarif, M., Bumstead, B., Buhse, M., & Kaczmarek, O. (2023). Multiple sclerosis and quality of life: The role of cognitive impairment on quality of life in people with multiple sclerosis. *Multiple Sclerosis and Related Disorders*. 10.1016/j.msard.2023.104966 37690436

[35] Gómez-Melero, S., Caballero-Villarraso, J., Escribano, B. M., Galvao-Carmona, A., Túnez, I., & Agüera-Morales, E. (2024). Impact of cognitive impairment on quality of life in multiple sclerosis patients—A. *Comprehensive Review Journal of Clinical Medicine*, *13*(11), 3321. 10.3390/jcm13113321 38893032 PMC11172944

[36] Berrigan, L. I., Fisk, J. D., Patten, S. B., Tremlett, H., Wolfson, C., Warren, S., Fiest, K. M., McKay, K. A., Marrie, R. A., & the CIHR Team in the Epidemiology and Impact of Comorbidity on Multiple Sclerosis. (2016). Health-related quality of life in multiple sclerosis: Direct and indirect effects of comorbidity. *Neurology*, *86*(15), 1417–1424. 10.1212/WNL.0000000000002564 26962068 PMC4831037

[37] Helvik, A. S., Bergh, S., Šaltytė Benth, J., Borza, T., Husebø, B., & Tevik, K. (2023). Pain and quality of life in nursing home residents with dementia after admission–a longitudinal study. *BMC Health Services Research*, *23*, 1032. 10.1186/s12913-023-10041-5 37759201 PMC10537464

[38] Huang, M. H., Tsai, C. F., Lin, Y. S., Kuo, Y. S., Hsu, C. C., & Fuh, J. L. (2024). A national survey on health-related quality of life for people with dementia in residential long-term care institutions. *Journal of the Formosan Medical Association*, *123*(7), 764–772. 38072742 10.1016/j.jfma.2023.11.012

[39] O’Brien, B. C., Harris, I. B., Beckman, T. J., Reed, D. A., & Cook, D. A. (2014). Standards for reporting qualitative research: A synthesis of recommendations. *Academic Medicine*, *89*(9), 1245–1251. 10.1097/ACM.0000000000000388 24979285

[40] Braun, V., Clarke, V., Boulton, E., Davey, L., & McEvoy, C. (2021). The online survey as a qualitative research tool. *International journal of social research methodology*, *24*(6), 641–654. 10.1080/13645579.2020.1805550

[41] Thomas, S L., Pitt, H., McCarthy, S., Arnot, G., & Hennessy, M. (2024). Methodological and practical guidance for designing and conducting online qualitative surveys in public health. *Health Promotion International*, *39*(3), daae061. 10.1093/heapro/daae061 38920273 PMC11200187

[42] Gyawali, B., Bagchi, S., Dakhil, Z., Yaseen, I., Cader, F. A., Pinto da Silva, L., Dewan, P., Sherifali, D., & Klassen, S. (2026). Exploring stakeholder perceptions of peer support initiatives in the management of diabetes in low-and middle-income countries: An online survey study. *PLOS Global Public Health*, *6*(2), e0005840. 10.1371/journal.pgph.0005840 41642908 PMC12875572

[43] Perfect, D., Griffiths, A. W., Da Silva, V., Lemos Dekker, M., McDermid, N., J., & Surr, C. A. (2021). Collecting self-report research data with people with dementia within care home clinical trials: Benefits, challenges and best practice. *Dementia*, *20*(1), 148–160. 10.1177/1471301219871168 31466468

[44] Fedorowicz, S., Dempsey, R. C., Ellis, N., & Gidlow, C. (2026). Hopeless but supported in that hopelessness: a qualitative study of how people experience talking to a GP about suicide. *Frontiers in Psychiatry*, *17*, 1744949. 10.3389/fpsyt.2026.1744949 41756568 PMC12933944

[45] Neate, S. L., Donald, A., Jelinek, G. A., & Nag, N. (2022). Experiences of and attitudes to lifestyle modification for the management of multiple sclerosis: A qualitative analysis of free-text survey data. *Health Expectations*, *25*(1), 214–222. 10.1111/hex.13364 34599857 PMC8849268

[46] Upadhyay, U. D., & Lipkovich, H. (2020). Using online technologies to improve diversity and inclusion in cognitive interviews with young people. *BMC Medical Research Methodology*, *20*(1), 159. 10.1186/s12874-020-01024-9 32539726 PMC7295690

[47] Carter, S. M., Shih, P., Williams, J., Degeling, C., & Mooney-Somers, J. (2021). Conducting qualitative research online: Challenges and solutions. *The Patient: Patient-Centered Outcomes Research*, *14*(6), 711–718. 10.1007/s40271-021-00528-w 34114170 PMC8192219

[48] Stalmeijer, R. E., Brown, M. E., & O’Brien, B. C. (2024). How to discuss transferability of qualitative research in health professions education. *The Clinical Teacher*, *21*(6), e13762. 10.1111/tct.13762 38497107

[49] Hancock, L. M., Galioto, R., Rhoads, T., Ontaneda, D., Nakamura, K., Ly, B., Krishnan, K., Miller, J. B., & Hua, L. H. (2024). Comparing cognitive profiles in older adults with multiple sclerosis and Alzheimer disease: More similarities than differences. *Neurology: Clinical Practice*, 14(4), e200327. 10.1212/CPJ.0000000000200327 PMC11152644 38846466

[50] Gold, S. M., Schulz, H., Mönch, A., Schulz, K. H., & Heesen, C. (2003). Cognitive impairment in multiple sclerosis does not affect reliability and validity of self-report health measures. *Multiple Sclerosis Journal*, *9*(4), 404–410. 10.1191/1352458503ms927oa 12926847

[51] Engel, L., Bailey, C., Bogatyreva, E., Batchelor, F., Devlin, N., Dow, B., Gilbert, A. S., Mulhern, B., Viney, R., & Peasgood, T. (2024). Appropriateness of the EQ-HWB for use in residential aged care: A proxy perspective. *The Patient-Patient-Centered Outcomes Research*, *17*(6), 673–683. 39235710 10.1007/s40271-024-00715-5 PMC11461598

[52] Health and Retirement Study (2021). Proxy selection in the Health and Retirement Study [Study documentation]. 10.7302/24833

[53] Phillips, J. M., Bickart, B. A., & Menon, G. (2025). Proxy reports of others’ behaviors: When are they more accurate? *Journal of Marketing Theory and Practice*, *33*(1), 29–42. 10.1080/10696679.2024.2385371

[54] Schmidt, S., Power, M., Green, A., Lucas-Carrasco, R., Eser, E., Dragomirecka, E., & Fleck, M. (2010). Self and proxy rating of quality of life in adults with intellectual disabilities: Results from the DISQOL study. *Research in Developmental Disabilities*, *31*(5), 1015–1026. 10.1016/j.ridd.2010.04.007 20478692

