# Supplementary material for: Informal carers’ perception on perspective taking when reporting as proxies about someone else’s health
Source: Qual Life Res. 2026 Aug 22;35(9):265. doi: 10.1007/s11136-026-04375-w (PMC13499833; doi:10.1007/s11136-026-04375-w)
Supplement: Supplementary file 1 [file 11136_2026_4375_MOESM1_ESM.docx]

**Table S1: Standards for Reporting Qualitative Research (SRQR)**

O’Brien B.C., Harris, I.B., Beckman, T.J., Reed, D.A., & Cook, D.A. (2014). Standards for reporting qualitative research: a synthesis of recommendations. *Academic Medicine, 89(9)*, 1245-1251.

| **No. Topic** | **Item** | **Page** |
| --- | --- | --- |
| **Title and abstract** |  |  |
| S1 Title | Concise description of the nature and topic of the study identifying the study as qualitative or indicating the approach (e.g., ethnography, grounded theory) or data collection methods (e.g., interview, focus group) is recommended | 1 |
| S2 Abstract | Summary of key elements of the study using the abstract format of the intended publication; typically includes objective, methods, results, and conclusions | 1-2 |
| **Introduction** |  |  |
| S3 Problem formulation | Description and significance of the problem/phenomenon studied; review of relevant theory and empirical work; problem statement | 3-5 |
| S4 Purpose or research question | Purpose of the study and specific objectives or questions | 4-5 |
| **Methods** |  |  |
| S5 Qualitative approach and research paradigm | Qualitative approach (e.g., ethnography, grounded theory, case study, phenomenology, narrative research) and guiding theory if appropriate; identifying the research paradigm (e.g., positivist, constructivist/interpretivist) is also recommended | 5-8 |
| S6 Researcher characteristics and reflexivity | Researchers’ characteristics that may influence the research, including personal attributes, qualifications/experience, relationship with participants, assumptions, or presuppositions; potential or actual interaction between researchers’ characteristics and the research questions, approach, methods, results, or transferability | 8 |
| S7 Context | Setting/site and salient contextual factors; rationale^a^ | 5-6 |
| S8 Sampling strategy | How and why research participants, documents, or events were selected; criteria for deciding when no further sampling was necessary (e.g., sampling saturation); rationale^a^ | 5-6 |
| S9 Ethical issues pertaining to human subjects | Documentation of approval by an appropriate ethics review board and participant consent, or explanation for lack thereof; other confidentiality and data security issues | 5 |
| S10 Data collection methods | Types of data collected; details of data collection procedures including (as appropriate) start and stop dates of data collection and analysis, iterative process, triangulation of sources/methods, and modification of procedures in response to evolving study findings; rationale^a^ | 8 |
| S11 Data collection instruments and technologies | Description of instruments (e.g., interview guides, questionnaires) and devices (e.g., audio recorders) used for data collection; if/how the instrument(s) changed over the course of the study | 5 |
| S12 Units of study | Number and relevant characteristics of participants, documents, or events included in the study; level of participation (could be reported in results) | 8 |
| S13 Data processing | Methods for processing data prior to and during analysis, including transcription, data entry, data management and security, verification of data integrity, data coding, and anonymization/deidentification of excerpts | 8 |
| S14 Data analysis | Process by which inferences, themes, etc., were identified and developed, including researchers involved in data analysis; usually references a specific paradigm or approach; rationale^a^ | 8 |
| S15 Techniques to enhance trustworthiness | Techniques to enhance trustworthiness and credibility of data analysis (e.g., member checking, audit trail, triangulation); rationale^a^ | 8 |
| **Results/Findings** |  |  |
| S16 Synthesis and interpretation | Main findings (e.g., interpretations, inferences, and themes); might include development of a theory or model, or integration with prior research or theory | 6-16 |
| S17 Links to empirical data | Evidence (e.g., quotes, field notes, text excerpts, photographs) to substantiate analytic findings | 9-16, Table S2 |
| **Discussion** |  |  |
| S18 Integration with prior work, implications, transferability, and contribution(s) to the field | Short summary of main findings; explanation of how findings and conclusions connect to, support, elaborate on, or challenge conclusions of earlier scholarship; discussion of scope of application/generalizability; identification of unique contribution(s) to scholarship in a discipline or field | 17-19 |
| S19 Limitations | Trustworthiness and limitations of findings | 19 |
| **Other** |  |  |
| S20 Conflicts of interest | Potential sources of influence or perceived influence on study conduct and conclusions; how these were managed | 20 |
| S21 Funding | Sources of funding and other support; role of funders in data collection, interpretation, and reporting | 21 |

^a^The rationale should briefly discuss the justification for choosing that theory, approach, method, or technique rather than other options available, the assumptions and limitations implicit in those choices, and how those choices influence study conclusions and transferability. As appropriate, the rationale for several items might be discussed together.

Table S2: Reasons for perceived most useful perspectives for communicating pain and physical function in people with dementia and MS symptom in people with MS

|  | **Perspectives** | **Reasons*** | **Illustrative Quotes** |
| --- | --- | --- | --- |
| MS symptom | Proxy-person | Familiarity with the person ^a, b^ | "*I am with my daughter a lot, so she communicates with me very well on how her symptoms are and how they affect her*" (P116, MS) |
|  |  | Person-first perspective ^a^ | *“Not sure if it [Proxy-person perspective] is more helpful, but seems more legit to centre the person living with MS*” (P 231, MS) |
|  | Proxy-proxy | Alternative objective insight ^a, b^ | *“I can provide an objective account of the patient's symptoms, uninfluenced by their personal biases or emotional state.”* (P140, MS).  *“By corroborating the patient's symptoms from my own observations, I can help reinforce their concerns and ensure their voice is heard.”* (P140, MS). |
|  |  | Carers able to add more observation on what the people with MS might miss ^a, b^ | *“Carers often notice subtle changes or patterns in the patient's behaviour, mood, or physical condition that the patient themselves might not be aware of or might downplay.”* (P140, MS).  *“I can offer context about the patient's daily life, environment, and habits, which can help healthcare professionals understand the symptoms' impact and potential triggers.”* (P140, MS). |
|  |  | Carers don’t think they can report from the proxy-person perspective ^a, b^ | *“I can't know what my partner is feeling but I can accurately describe what I see.”* (P247, MS)  *“Because all you can do is give your account of the situation. You cannot feel or explain how someone feels that has MS unless you have it.”* (P242, MS) |
|  |  | Additional insights and usefulness of added perspective ^a, b^ | *“Carers may pick up on nonverbal cues, such as body language or tone of voice, that can provide additional insights into the patient's condition.”* (P140, MS).  *“Because they're already receiving her perspective so me imagining her perspective won't add anything and won't be as accurate anyway. Whereas an outside perspective could be helpful.”* (P79, MS) |
|  | Either | Complementary view ^a^ | *“Considering the individuals experience and viewpoint is the most important. But I believe also speaking from an outside perspective is important ...”* (P184, MS) |
|  |  | Familiarity with the person ^a, b^ | *“I am with my daughter a lot and she communicate well with me about how she feels so I can communicate both ways”* (P116, MS)  *“I do take daily notes of the client and their experiences and difficulties they may be having. I can often recognise some warning signs of the persons tiredness and ability to do various tasks; we try not to cram to many activities in the day i.e. only 1 appointment and taking her wheelchair out if we are out for a longer time. I am very close to the clients age and the person often says, "you know me so well"* (P204, MS) |
| Dementia- Pain | Proxy-person | Familiarity with the person ^a, b^ | *"I knew my mother very well"* (P25, Dementia) *"I knew my mother well and was able to put myself in her shoes…"* (P32, Dementia) |
|  |  | Helps the person feel included ^a^ | *"Able to make patient feel useful and needed"* (P43, Dementia) *"They [people with Dementia] need to feel as part of the present and not to ignore them"* (P51, Dementia) |
|  |  | Encourages the carers to be more engaged ^b^ | " *It* *[proxy-person perspective] assists you to exert more effort in helping to master basic things*" (P51, Dementia) |
|  |  | Person-first perspective ^a^ | "…*Her experience of pain rather than mine was most relevant*" (P32, Dementia) "*Consider patients' needs to be more important than your own"* (P45, Dementia) |
|  | Proxy-proxy | Helps to convey alternative objective insight ^b, c^ | *“Because you see what they can't or won't …”* (P40, Dementia)  *“We do not know what the person is thinking”* (P46, Dementia) |
|  |  | Familiarity with the person ^a, b^ | *“Close long-term relationship, could only imagine what Dad thought and felt when he was bedridden and not talking much, or sense.”* (P30, Dementia). |
|  |  | Helps to effectively communicate with HCPs ^b, c^ | *“Because I believe I can explain her pain in a way that her Doctor believes and doesn't trivialise.”* (P2, Dementia).  “*I can potentially quote Dad using his words to help them find the source of the pain*.” (P3, Dementia) |
|  | Either | Balanced perspective ^b^ | *“If I answer from my perspective as observer, I may interpret her pain incorrectly so if I also imagine it in her shoes, I could get a more balanced view”* (P35, Dementia)*.*  *“These are more empathetic / humane approaches, plus cover both areas of experience and understanding of the client”* (P36, Dementia) |
|  |  | Familiarity with the person ^a, b^ | *“Probably only because I know my Dad better than most people.”* (P13, Dementia)  *“By knowing how they were previously’’* (P22, Dementia) |
|  |  | Communication and observation ^b,c^ | *“Imagining the pain from Mum's perspective helps me to properly acknowledge her pain and give it a voice. Then relaying to the health care provider (HCP) my observations help clarify the picture. This process helps Mum feel heard and believed, while also relaying the true situation to the healthcare professionals without undermining Mum.”* (P11, Dementia)  *“He is feeling the pain I am observing his changed behaviour in relation to pain”* (P28, Dementia) |
| Dementia- Physical function | Proxy-person | Person-first perspective ^a^ | "*They [proxy-person perspectives] are best because they are patient-centred*" (P51, Dementia)  *"…Putting myself in my partner's shoes helps to see it from his perspective…"* (P24, Dementia) |
|  | Proxy-proxy | Avoid conflict ^c^ | *“Dad could be belligerent. Strongly opinionated... He would get angry if I answered for him or on his behalf*.” (P30, Dementia) |
|  |  | Familiarity with the person ^a, b^ | “*I have known my wife for a very long time and can see the physical changes.”* (P22, Dementia) |
|  |  | Alternative objective insight ^c^ | *“Keeping an objective perspective on the provided Data, collected from the client's symptoms, will be more accurate findings for communication.”* (P36, Dementia) |
|  |  | Carers don’t think they can report from the proxy-person perspective ^b, c^ | *“I lack the understanding of what being in Dad's shoes would be like.”* (P3, Dementia)  *“I've tried to imagine what my husband's experience ... I can't do it…”* (P14, Dementia) |
| *The reasons and categories were developed from proxies’ responses. Categories: ^a^ Person-centred, ^b^ Proxy-centred and ^c^ Communication centred reasons | | | |
